# Real-World Adherence and Follow-Up Colonoscopy After Multitarget Stool DNA Screening in a Regional Healthcare System

**DOI:** 10.36469/001c.168717

**Published:** 2026-09-09

**Authors:** Amanda Hanson, Shrey Gohil, Ayman Abdelmoity, Mohamed Omar, Adam Kazberuk, Jordan Karlitz, Mallik Greene

**Affiliations:** 1 Precision Medicine Spartanburg Regional Healthcare System; 2 Medical Affairs Abbott

**Keywords:** colorectal neoplasms, early detection of cancer, colonoscopy, cancer screening, healthcare disparities, multitarget stool DNA test, patient compliance

## Abstract

**Background:**

Multitarget stool DNA (mt-sDNA) testing is an established noninvasive colorectal cancer (CRC) screening option for average-risk adults, but real-world completion of the screening cascade through colonoscopy remains incompletely characterized.

**Objectives:**

This study aimed to evaluate mt-sDNA adherence, test positivity, and follow-up colonoscopy completion in a regional healthcare system and identify factors associated with screening cascade completion.

**Methods:**

This retrospective cohort study analyzed mt-sDNA test kits ordered for average‑risk, screening‑eligible individuals in the Spartanburg Regional Healthcare System (South Carolina, USA) from January 2016 to June 2023. Analyses were conducted at the test-kit level; patients could contribute more than one eligible test during the study period. Eligible tests were from patients aged 45 to 85 years with ≥6 months of prior continuous insurance coverage and no high-risk CRC conditions. Outcomes were adherence (kit return within 365 days), test positivity among adherent test kits, and follow‑up colonoscopy within 365 days among eligible positive cases. Multivariable logistic regression and Cox proportional hazards regression models evaluated factors associated with adherence and time to colonoscopy.

**Results:**

Of 8439 included test kits, 5733 (67.9%) were returned within 1 year. Among returned test kits, 1018 (17.8%) yielded a positive mt-sDNA result. Of 853 positive tests eligible for follow-up assessment, 596 (69.9%) were for patients who completed follow-up colonoscopy. Adherence to mt-sDNA testing was associated with non-Medicaid insurance, higher income, digital outreach, prior test return, and lower comorbidity burden. Time to colonoscopy was shorter for patients in the middle vs lowest income group.

**Discussion:**

Medicaid insurance was the strongest insurance-related predictor of nonadherence, with rates approximately 20 percentage points below those with commercial or Medicare-type coverage. Digital outreach and prior test return history were among the independent predictors of adherence. Income was the primary independent predictor of time to follow-up colonoscopy, potentially reflecting financial barriers to diagnostic follow-up.

**Conclusions:**

In this real-world cohort study, approximately two-thirds of tests were returned and 70% of eligible positive tests completed follow-up colonoscopy within 1 year. These findings identify potentially modifiable socioeconomic and outreach-related factors associated with screening cascade completion and support targeted interventions for lower-income and Medicaid-insured populations.

## BACKGROUND

Colorectal cancer (CRC) remains a leading cause of cancer-related morbidity and mortality in the United States (US), despite the availability of effective screening strategies that can detect precancerous lesions and early-stage disease.^1,2^ In 2026, CRC is projected to account for approximately 158 850 new diagnoses and 55 230 deaths in the US, making it the second leading cause of cancer-related mortality in men and women combined.^1^ Sustained reductions in CRC incidence and mortality over recent decades are attributable in part to the wider adoption of population-based screening programs.^2,3^ Despite this progress, screening uptake remains below national targets; in 2023, approximately 63.5% of eligible US adults were current with recommended screening,^4^ well short of the 80% goal set by the American Cancer Society National Colorectal Cancer Roundtable.^5^

Several modalities are currently recommended for CRC screening, including direct visualization strategies such as colonoscopy and stool-based options including fecal immunochemical testing (FIT) and multitarget stool DNA (mt-sDNA) testing.^6^ Stool-based tests can be completed at home without bowel preparation or sedation, making them an accessible option for patients unable or reluctant to undergo colonoscopy.^6^ Among available stool-based options, mt-sDNA testing detects aberrant methylation and mutant *KRAS* DNA markers as well as fecal hemoglobin, and has demonstrated higher sensitivity for CRC and advanced adenomas compared with FIT.^7–9^ Comparative studies have found that patients are more likely to participate in mt-sDNA screening compared with FIT.^10,11^

Although mt-sDNA testing has demonstrated higher sensitivity for CRC and advanced adenomas than FIT in comparative studies, its real-world effectiveness depends on completion of the full screening cascade, including test kit return, identification of positive results, and timely follow-up colonoscopy among patients with a positive test.^12–15^ Prior studies of real-world practice have demonstrated that patients are more likely to complete follow-up colonoscopy after a positive mt-sDNA result than after a positive FIT.^10,12,16,17^

Although mt-sDNA performance characteristics are well established, less is known about where attrition occurs across the real-world screening continuum and which patient- and system-level factors are associated with incomplete follow-through.^18^ In this context, patient-level factors refer to individual demographic and clinical characteristics (eg, age, insurance type, comorbidity burden), whereas system-level factors refer to modifiable, health system–controlled variables such as outreach modality and ordering provider specialty. Real-world evidence from large health systems can provide critical insights into how mt-sDNA performs outside controlled settings, where insurance coverage, digital engagement, comorbidity burden, and prior screening experience may shape patient behavior.^12,14^ Few real-world studies have simultaneously evaluated adherence, test positivity, and follow-up colonoscopy completion within a single integrated healthcare system. Such analyses can help identify modifiable points of attrition and inform targeted quality improvement at the health system level.^19,20^

In this retrospective cohort study, we evaluated real-world mt-sDNA screening adherence, test positivity, and follow-up colonoscopy completion within a regional healthcare system and identified patient- and system-level factors associated with screening completion across the care continuum.

## METHODS

### Data Source

This retrospective cohort study linked mt-sDNA laboratory and order data from Abbott (Madison, Wisc.) with administrative healthcare claims data from the Komodo Health Healthcare Map® database. The analysis focused on patients associated with Spartanburg Regional Healthcare System, a large regional integrated healthcare delivery network serving Upstate South Carolina that includes 5 hospitals, more than 120 physician practices and outpatient locations, and approximately 1400 affiliated providers. Spartanburg Regional Healthcare System was selected because mt-sDNA screening adherence and follow-up colonoscopy outcomes had not previously been evaluated within this health system, providing an opportunity to characterize real-world screening performance through the use of linked laboratory and claims data. Patients were attributed to Spartanburg Regional Healthcare System based on ordering provider and facility identifiers, healthcare utilization within the Spartanburg network, and health system attribution algorithms available within the linked dataset. The Abbott database contains laboratory and order information from more than 20 million mt-sDNA tests processed nationally, including test shipment and result dates, ordering provider and facility information, patient demographics, and outreach-related variables. The Komodo Health Healthcare Map® database contains longitudinal medical and pharmacy claims from a large, geographically diverse population of insured individuals across the US. For this analysis, shipment records for commercially available mt-sDNA tests ordered between January 2016 and June 2023 were linked to claims data using a privacy-preserving tokenization approach. The linked dataset enabled longitudinal assessment of screening participation, mt-sDNA test results, and follow-up colonoscopy utilization among insured patients with observable healthcare encounters during the study period. This methodology allowed patient-level integration of screening and downstream healthcare events while maintaining compliance with the Health Insurance Portability and Accountability Act (HIPAA). In accordance with 45 CFR § 46.104(d)(4), this secondary analysis of existing de-identified data was exempt from institutional review board oversight and did not require informed consent. The study was conducted in accordance with the Declaration of Helsinki.

### Study Population

**Figure 1.**
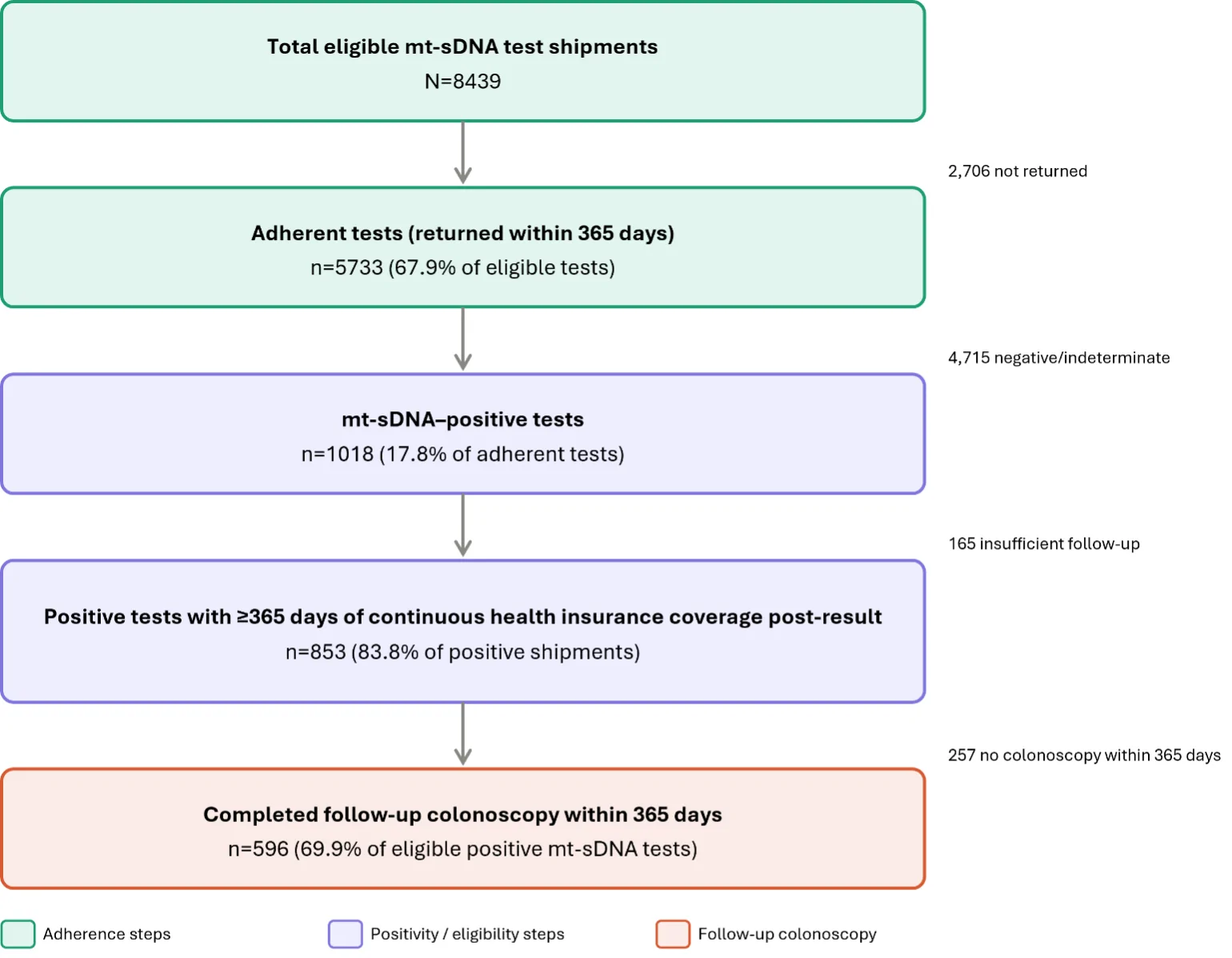
Screening Cascade of mt-sDNA Testing and Follow-up Colonoscopy Among Eligible Test Kits Abbreviation: mt-sDNA, multitarget stool DNA. Cascade depicts sequential outcomes for the 8439 eligible test kits: adherence (kit return within 365 days), test positivity (among adherent test kits), and follow-up colonoscopy completion within 365 days (among eligible positive test kits with ≥365 days of post-result continuous health insurance coverage). Percentages are calculated relative to the preceding cascade step.

The source population comprised 26 163 mt-sDNA test kit requests recorded at Spartanburg Regional Healthcare System during the study period. Sequential eligibility screening was applied to identify kits ordered for average-risk, screening-eligible individuals (**Figure 1**). Test kit requests were excluded if the associated patient was outside the eligible age range at the shipment date (<45 or >85 years) or did not have ≥6 months of continuous health plan enrollment prior to the shipment date. The study focused on individuals at average risk for CRC, consistent with the population for whom mt-sDNA is indicated for routine CRC screening. Individuals were excluded if available claims before the index mt-sDNA shipment indicated conditions associated with increased CRC risk, including a personal history of CRC or colorectal adenomas/polyps, inflammatory bowel disease, a family history of CRC or polyps, or hereditary CRC syndromes or genetic susceptibility.

Analyses were performed at the shipment level because the objective was to evaluate adherence across real-world screening opportunities rather than unique patients. Although some patients contributed multiple shipments during the study period, 92.9% of patients contributed only one shipment, and the mean number of shipments per patient was 1.07. Therefore, the potential impact of within-patient correlation on the overall findings is expected to be minimal. Nevertheless, because repeat shipments from the same patient are not statistically independent, some correlation between observations may remain and should be considered when interpreting the results. Prior mt-sDNA return history was defined using an existing laboratory database variable indicating whether a patient had previously returned an mt-sDNA test. Because prior return history was included as a covariate in both descriptive and multivariable analyses, the primary analysis included all eligible shipments rather than being restricted to first-time test orders. This approach was selected to evaluate real-world screening behavior across sequential screening opportunities during the study period. Continuous health insurance enrollment was required to confirm study eligibility and ensure sufficient observability of healthcare encounters during follow-up.

### Patient Characteristics and Study Outcomes

Baseline demographic characteristics in this analysis included age group, sex, race/ethnicity, insurance type, median annual household income by ZIP code, provider specialty, measures of patient outreach modality (full digital reminders, email only, short messaging service [SMS] text only, or no digital reminders), prior mt-sDNA testing history, and Charlson Comorbidity Index (CCI). CCI was calculated using diagnosis codes from claims data. Conditions were required to meet standard claims-based confirmation criteria (≥1 inpatient claim or ≥2 outpatient claims separated by >30 days), and CCI was categorized as 0, 1, or ≥2.

Three sequential cascade outcomes were assessed: mt-sDNA kit adherence, test positivity, and follow-up colonoscopy completion. Adherence was defined as return and processing of the mt-sDNA kit within 365 days of the shipment date and was assessed among all eligible test kits. The 365-day window was selected as a standard real-world evidence ascertainment period that allows sufficient time to capture delayed kit completion. It also aligns with the 365-day follow-up colonoscopy endpoint used elsewhere in this study and with adherence windows used in prior real-world mt-sDNA studies.^21–23^ Test positivity was assessed among adherent kits and defined as receipt of a positive mt-sDNA result. For descriptive analyses, positivity was evaluated among all adherent test kits; however, analyses involving follow-up colonoscopy were restricted to positive tests with ≥365 days of continuous post-result health insurance enrollment. Follow-up colonoscopy was defined as completion of a qualifying colonoscopy procedure within 365 days of the positive mt-sDNA result date, as identified from administrative claims records. Both screening and diagnostic colonoscopy procedures meeting study criteria were considered qualifying follow-up examinations.

Covariates assessed in relation to cascade outcomes included: age at shipment (45-49, 50-64, 65-74, and 75-85 years); sex; race and ethnicity; health insurance type (commercial, Medicaid, Medicare Advantage, and Traditional Medicare); median annual household income estimated from ZIP code in US dollars (<$50 000; $50 000 to <$75 000; ≥$75 000); ordering provider specialty (gastroenterologist, obstetrician/gynecologist, primary care physician, nurse practitioner/physician assistant); type of patient outreach; prior mt-sDNA return history; and CCI score.

### Statistical Analysis

Patient and test characteristics were summarized using frequencies and percentages for categorical variables. Comparisons of categorical outcomes across subgroups were evaluated using chi-square tests. Variables with missing or unknown data were analyzed as separate categories where applicable. Because test positivity reflects underlying disease prevalence rather than screening behavior, multivariable regression analyses focused on adherence and follow-up colonoscopy outcomes.

Factors independently associated with adherence to mt-sDNA testing were identified using multivariable logistic regression, with results reported as adjusted odds ratios (aORs) with 95% confidence intervals (CIs) and *P* values. Kaplan-Meier methods were used to evaluate time to mt-sDNA adherence, stratified by outreach type and prior return history, with differences assessed using the log-rank test.

Time to follow-up colonoscopy was analyzed using multivariable Cox proportional hazards regression among eligible positive tests, with time measured from the positive mt-sDNA result date to the date of the first qualifying colonoscopy. Cox regression was selected to account for variation in timing of follow-up colonoscopy and censoring among patients without documented colonoscopy within 365 days. Patients without a qualifying colonoscopy during follow-up were censored at 365 days after the positive mt-sDNA result date. Results are reported as adjusted hazard ratios (aHRs) with 95% CIs and *P* values. aHRs >1 indicate a higher rate of follow-up colonoscopy completion over time.

All statistical analyses were performed using SAS software (version 9.4; SAS Institute). All analyses were conducted using two-sided hypothesis testing, and a 2-sided *P* value <.05 was considered statistically significant. This study was reported in accordance with the Strengthening the Reporting of Observational Studies in Epidemiology (STROBE) reporting guideline.

## RESULTS

### Study Population

Of 26 163 mt-sDNA test kit requests recorded at Spartanburg Regional Healthcare System between January 2016 and June 2023, 10 were excluded for patient age <45 or >85 years, 17 030 for lack of ≥6 months of continuous health insurance coverage prior to shipment, and 684 for evidence of high-risk CRC conditions prior to shipment, yielding a final cohort of 8439 average-risk, screening-eligible mt-sDNA test kits. These 8439 shipment observations were contributed by 7863 unique patients. Most patients (92.9%) contributed only one shipment during the study period, while 7.1% contributed more than one, corresponding to a mean of 1.07 shipments per patient. Most exclusions were attributable to insufficient continuous health insurance enrollment prior to shipment (n = 17 030; 65.1%), followed by exclusion for high-risk CRC conditions (n=684) and age ineligibility (n=10). Among the 8439 eligible test kits, 5733 (67.9%) were returned and processed within 365 days of shipment (**Figure 1**). Of these returned tests, 1018 (17.8%) yielded a positive mt-sDNA result. Among positive tests, 853 (83.8%) had ≥365 days of continuous post-result health insurance enrollment and were eligible for follow-up colonoscopy assessment; 596 of these patients (69.9%) completed follow-up colonoscopy within 365 days (**Figure 1**). In a sensitivity analysis that included all positive mt-sDNA tests regardless of post-result continuous insurance enrollment, the observed follow-up colonoscopy completion rate was 68.2% (694/1018) (**Supplementary Table S1**), demonstrating results similar to the primary analysis.

Among eligible test kits, patients were most commonly aged 50-64 years (45.1%), female (56.8%), White (59.2%), with a CCI score of 0 (69.9%), and residing in ZIP codes with median annual household income of $50 000 to <$75 000 (58.9%) (**Table 1**). Medicare Advantage accounted for the majority of tests (57.7%) and most were ordered by primary care physicians (78.7%). Approximately half of all tests (50.9%) were linked to SMS (text message)-only outreach, and most patients had not previously returned an mt-sDNA kit (87.0%).

**Table 1.** Characteristics of mt-sDNA Test Kits and Outcomes Across the Colorectal Cancer Screening Cascade^a^

	**Total Eligible Test Kits, n (%)**	**Adherence, n (% of Total Eligible Test Kits)**	**Eligible Positive Test Kits, n (% of Adherent Test Kits)^b^**	**Follow-up Colonoscopy, n (% of Eligible Positive Test Kits)**
Overall	8439 (100)	5733 (67.9)	853 (14.9)	596 (69.9)
Age, years				
*P* value		<.001	<.001	.256
45-49	506 (6.0)	336 (66.4)	16 (4.8)	14 (87.5)
50-64	3807 (45.1)	2433 (63.9)	279 (11.5)	186 (66.7)
65-74	3182 (37.7)	2266 (71.2)	408 (18.0)	290 (71.1)
75-85	944 (11.2)	698 (73.9)	150 (21.5)	106 (70.7)
Sex				
*P* value		.417	.004	.344
Female	4791 (56.8)	3272 (68.3)	469 (14.3)	334 (71.2)
Male	3648 (43.2)	2461 (67.5)	384 (15.6)	262 (68.2)
Race/ethnicity				
*P* value		<.001	<.001	.013
White	4998 (59.2)	3445 (68.9)	630 (18.3)	424 (67.3)
Black	1552 (18.4)	976 (62.9)	129 (13.2)	96 (74.4)
Other/unknown	1889 (22.4)	1312 (69.5)	94 (7.2)	76 (80.9)
CCI				
*P* value		<.001	<.001	.736
0	5895 (69.9)	4126 (70.0)	564 (13.7)	397 (70.4)
1	1292 (15.3)	825 (63.9)	143 (17.3)	96 (67.1)
≥2	1252 (14.8)	782 (62.5)	146 (18.7)	103 (70.6)
Median annual household income by ZIP code, $				
*P* value		<.001	<.001	.010
<50 000	1830 (21.7)	1124 (61.4)	183 (16.3)	113 (61.8)
50 000 to <75 000	4971 (58.9)	3437 (69.1)	509 (14.8)	374 (73.5)
≥75 000	1638 (19.4)	1172 (71.6)	161 (13.7)	109 (67.7)
Health insurance type				
*P* value		<.001	<.001	.430
Commercial	2880 (34.1)	1979 (68.7)	163 (8.2)	120 (73.6)
Medicaid	577 (6.8)	288 (49.9)	36 (12.5)	26 (72.2)
Medicare Advantage	4866 (57.7)	3380 (69.5)	638 (18.9)	441 (69.1)
Traditional Medicare	116 (1.4)	86 (74.1)	16 (18.6)	9 (56.3)
Provider specialty				
*P* value		.001	<.001	.312
Gastroenterologist	79 (0.9)	55 (69.6)	15 (27.3)	11 (73.3)
NP/PA	1463 (17.3)	926 (63.3)	142 (15.3)	94 (66.2)
OB/GYN	94 (1.1)	66 (70.2)	5 (7.6)	5 (100)
Other	162 (1.9)	114 (70.4)	16 (14.0)	9 (56.3)
Primary care physician	6641 (78.7)	4572 (68.9)	675 (14.8)	477 (70.7)
Type of outreach				
*P* value		<.001	<.001	.369
Full digital	136 (1.6)	114 (83.8)	14 (12.3)	12 (85.7)
No digital	1549 (18.4)	902 (58.2)	130 (14.4)	84 (64.6)
Partial digital – email only	31 (0.4)	28 (90.3)	5 (17.9)	3 (60.0)
Partial digital – SMS only	4296 (50.9)	2868 (66.8)	356 (12.4)	247 (69.4)
Unknown	2427 (28.8)	1821 (75.0)	348 (19.1)	250 (71.8)
Patient return history				
*P* value		<.001	<.001	.308
New patient	7342 (87.0)	4868 (66.3)	739 (15.2)	521 (70.5)
≥1 prior returns	1097 (13.0)	865 (78.9)	114 (13.2)	75 (65.8)

Abbreviations: CCI, Charlson Comorbidity Index; mt-sDNA, multitarget stool DNA; NP/PA, nurse practitioner/physician assistant; OB/GYN, obstetrician/gynecologist; SMS, short messaging service.

^a^Adherence percentages are calculated among all eligible mt-sDNA test kits within each subgroup. Test positivity percentages are calculated among adherent test kits within each subgroup; numerators are restricted to test kits with a positive mt-sDNA result and ≥365 days of continuous health insurance coverage following the positive result date. Follow-up colonoscopy percentages are calculated among positive test kits with ≥365 days of continuous health insurance coverage following the positive result date. *P*-values were derived from chi-square tests comparing proportions across subgroups.

### Testing Adherence

**Figure 2.**
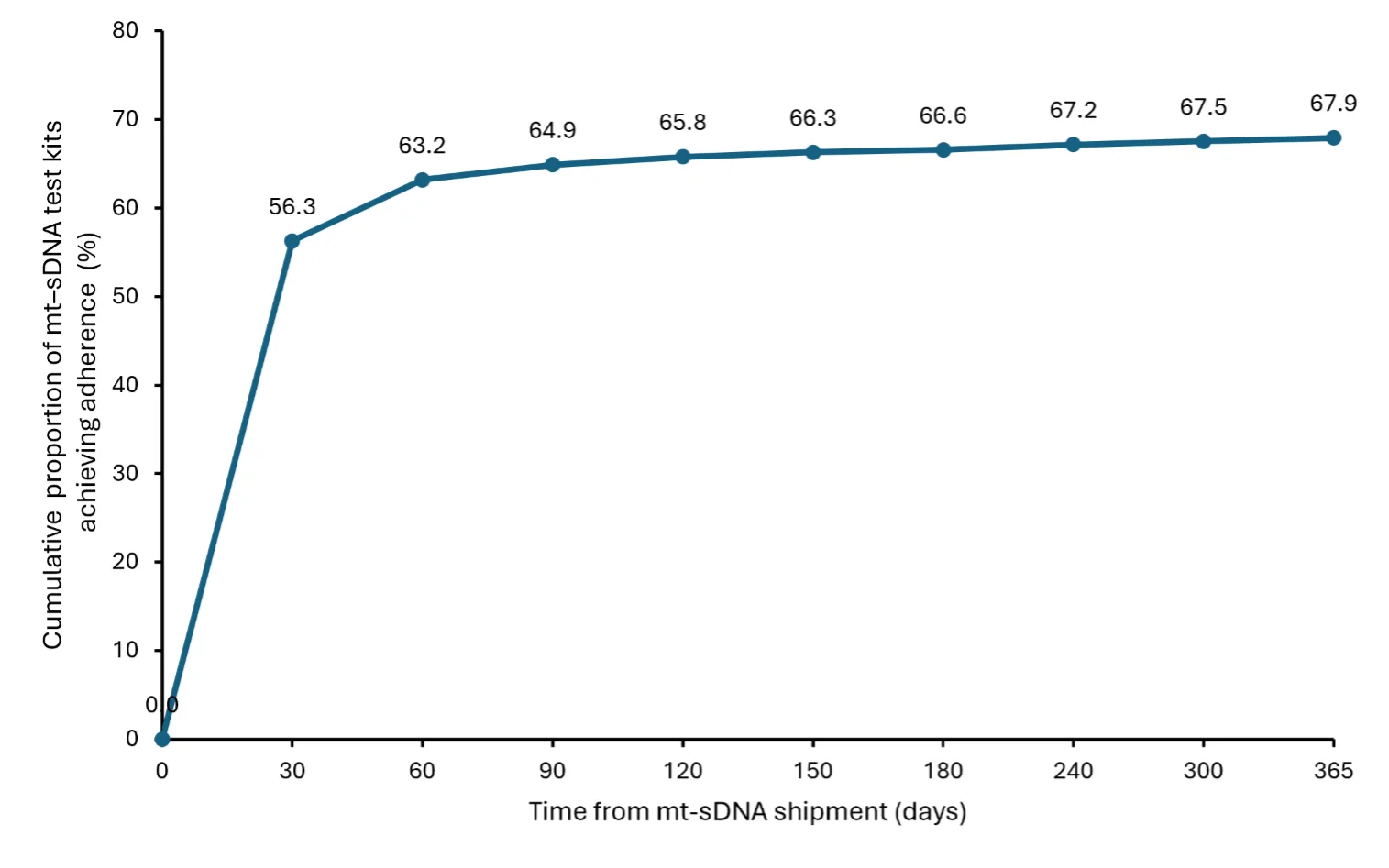
Cumulative Proportion of mt-sDNA Test Kits Achieving Adherence Within 365 Days of Shipment Abbreviation: mt-sDNA, multitarget stool DNA. Cumulative percentage reflects the proportion of test kits that achieved adherence (test return) within each time interval after the shipment date. One test kit is excluded due to missing information on adherence timing (n=5732 included).

Adherence rates varied significantly across all covariate subgroups except sex (*P* = .417). Rates were highest among patients aged 75-85 years (73.9%), those with traditional coverage (74.1%), those in the highest household income group (≥$75 000; 71.6%), and those receiving full digital (83.8%) or email-only outreach (90.3%; n = 31 tests). Adherence was lowest among Medicaid-insured patients (49.9%) and those with no digital outreach (58.2%). Patients with at least one prior mt-sDNA return had significantly higher adherence than new patients (78.9% vs 66.3%; *P* < .001) (**Table 1**). Most kits were returned within the first 30 days after shipment, during which cumulative adherence reached 56.3%; cumulative adherence reached 67.9% by 365 days (**Figure 2**). Among returned test kits, descriptive positivity rates increased with age, comorbidity burden, and were higher in Medicare Advantage and traditional Medicare patients than in those with commercial insurance (**Table 1**).

**Figure 3.**
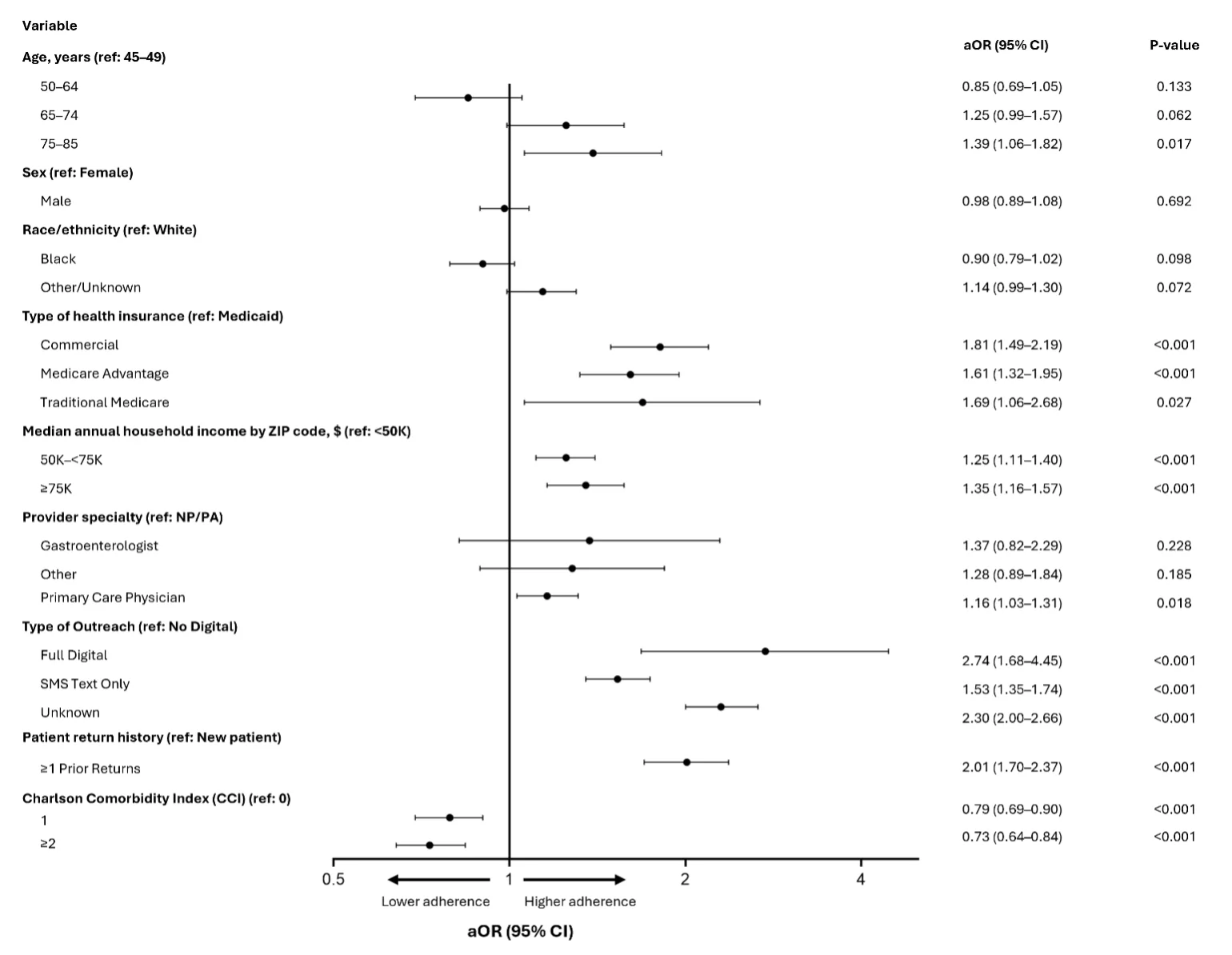
Multivariable Logistic Regression Analysis of Factors Associated with Adherence to mt-sDNA Testing Abbreviations: aOR, adjusted odds ratio; CCI, Charlson Comorbidity Index; CI, confidence interval; mt-sDNA, multitarget stool DNA; NP/PA, nurse practitioner/physician assistant; OB/GYN, obstetrician/gynecologist; SMS, short messaging service. Categories excluded from the regression model due to sparse observations: OB/GYN (provider specialty; n = 94 total test kits, n = 5 positive tests) and partial digital outreach – email only (outreach type; n = 31 total test kits).

In multivariable logistic regression analyses evaluating adherence (**Figure 3**), greater comorbidity burden was associated with lower adherence (CCI 1: aOR, 0.79; CCI ≥2: aOR, 0.73; both *P* < .001; reference: CCI 0). Higher adherence was independently associated with older age (75-85 years: aOR, 1.39; *P* = .017; reference: 45-49 years), middle and higher household income ($50 000 to <$75 000: aOR, 1.25; ≥$75 000: aOR, 1.35; both *P* < .001; reference <$50 000), all non-Medicaid insurance types (commercial: aOR, 1.81; Medicare Advantage: aOR, 1.61; Traditional Medicare: aOR, 1.69; all *P* < .05; reference: Medicaid), primary care physician (aOR, 1.16; *P* = .018; reference: nurse practitioner/physician assistant), full or partial digital outreach (full digital: aOR, 2.74; SMS only: aOR, 1.53; both *P* < .001; reference: no digital), and prior test return history (aOR, 2.01; *P* < .001; reference: new patient).

**Figure 4.**
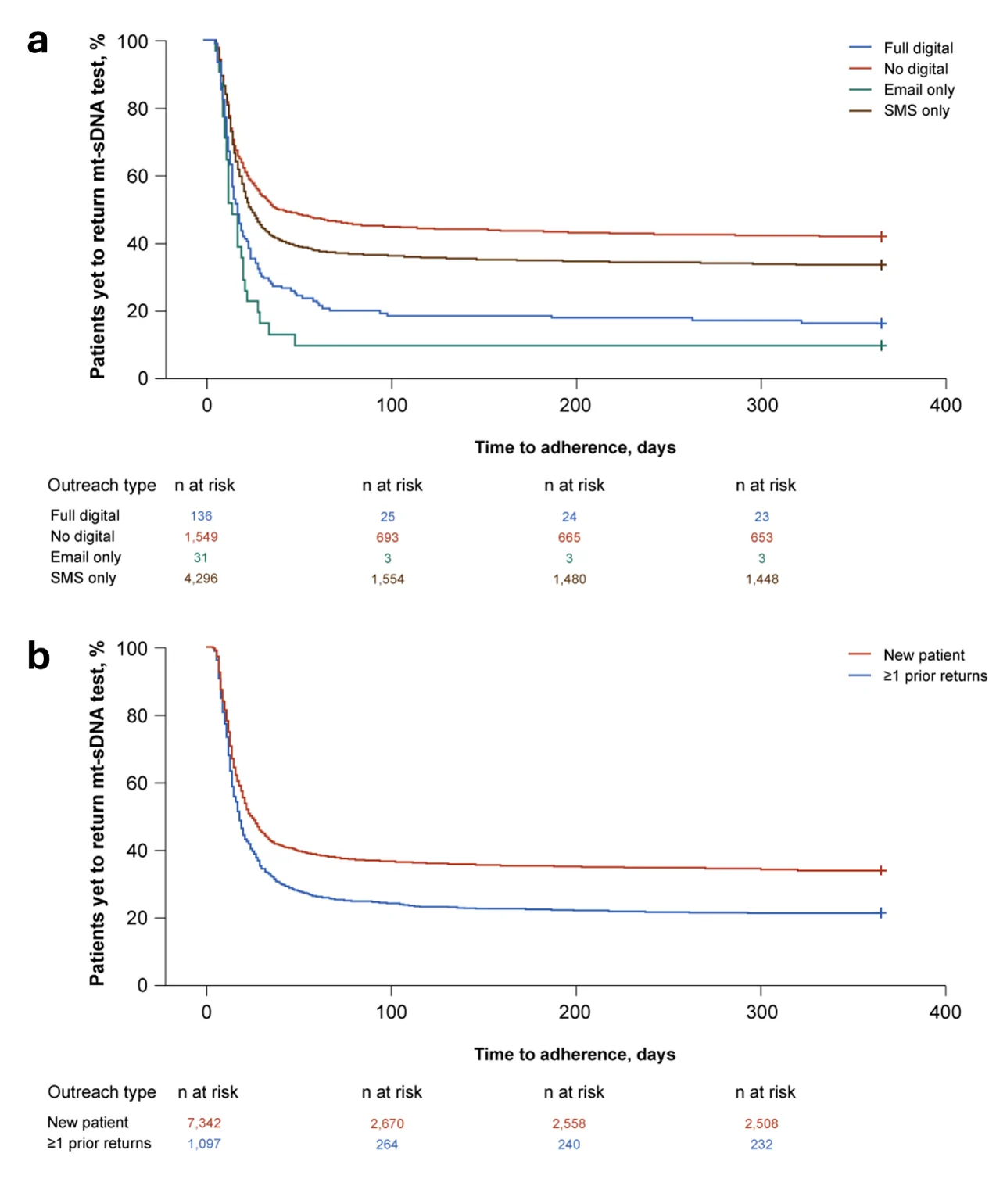
Time to mt-sDNA Test Adherence (**a**) by Outreach Type (Log-Rank Test: χ^2^ = 185.19, *df* = 4, *P* < .0001) and (**b**) by Prior Test Return History (Log-Rank Test: χ^2^= 76.48, *df* = 1, *P* < .0001) Abbreviations: mt-sDNA, multitarget stool DNA; SMS, short messaging service. Curves depict the cumulative proportion of test kits achieving adherence over time.

Kaplan-Meier curves illustrating time to adherence by outreach type and prior return history are presented in **Figure 4**; log-rank tests confirmed significant differences between strata for both outreach type (*P* < .0001) and prior return history (*P* < .0001).

### Follow-up Colonoscopy

Among eligible mt-sDNA positive tests, follow-up colonoscopy rates differed significantly by race/ethnicity (*P* = .013), with the highest completion in the other/unknown group (80.9%) and the lowest among White patients (67.3%), and by household income (*P* = .010), with the lowest rate in the <$50 000 group (61.8%) compared with the $50 000 to <$75 000 group (73.5%). Rates did not differ significantly by age, sex, insurance type, provider specialty, outreach type, prior return history, or comorbidity (all *P* < .05) (**Table 1**).

**Figure 5.**
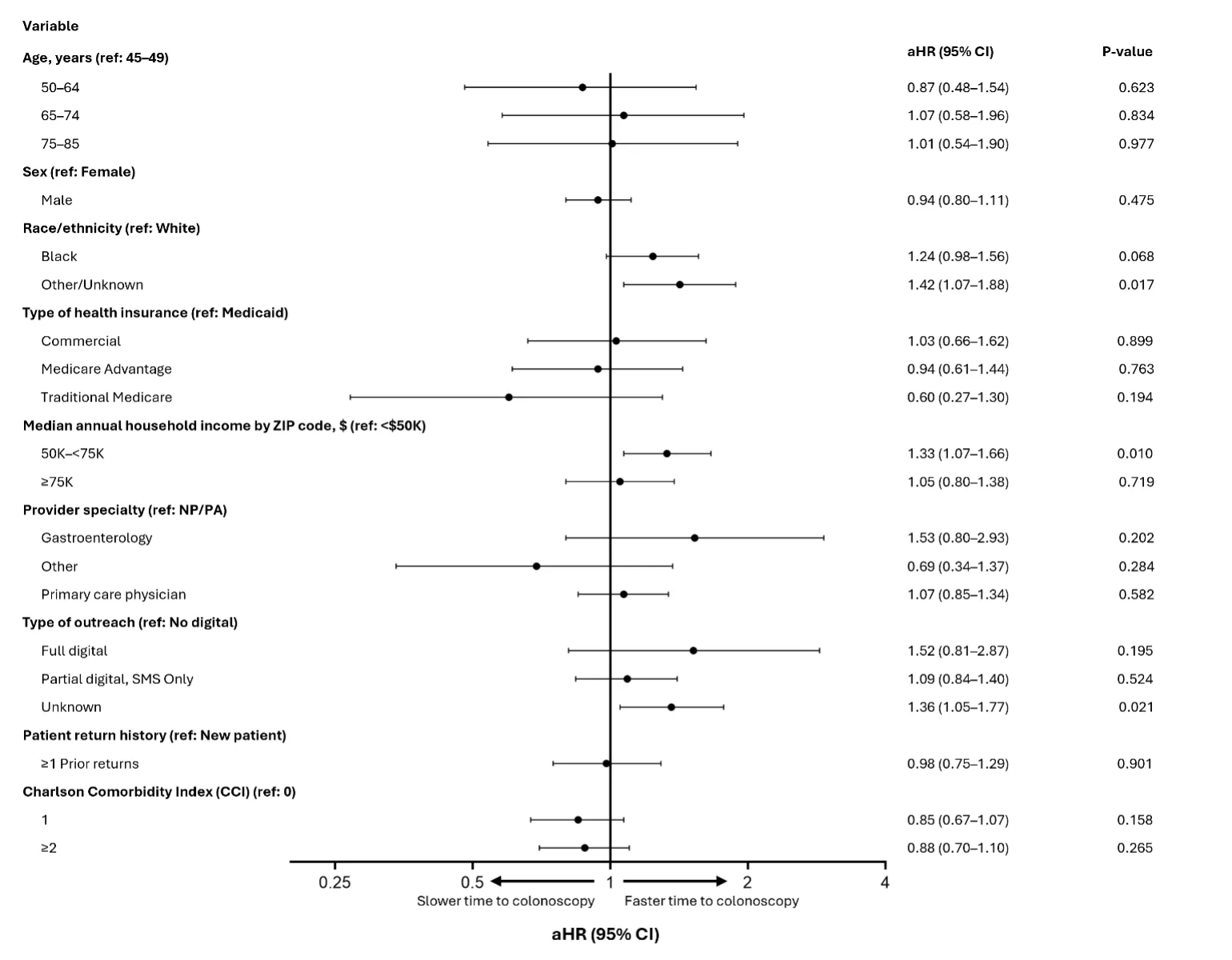
Factors Associated with Time to Follow-up Colonoscopy Among mt-sDNA–Positive Test Kits. Multivariable Cox Proportional Hazards Regression Analysis Abbreviations: aHR, adjusted hazard ratio; CCI, Charlson Comorbidity Index; CI, confidence interval; mt-sDNA, multitarget stool DNA; NP/PA, nurse practitioner/physician assistant; OB/GYN, obstetrician/gynecologist; SMS, short messaging service. Categories excluded from the regression model due to sparse observations: OB/GYN (n = 5 events) and partial digital outreach – email only (n = 3 events).

In Cox proportional hazards regression (**Figure 5**), the significant independent predictors of time to follow-up colonoscopy were household income and race/ethnicity. Patients in the middle-income category completed colonoscopy at a faster rate than those in the lowest income group (aHR, 1.33, 95% CI, 1.07-1.66; *P* = .010), and the other/unknown race/ethnicity group showed significantly faster time to follow-up than White patients (aHR, 1.42; 95% CI, 1.07-1.88; *P* = .017). Because this category combines multiple racial and ethnic groups as well as records with incomplete demographic information, these findings should be interpreted cautiously and are not readily attributable to a specific population subgroup.

## DISCUSSION

This retrospective cohort study characterized the real-world mt-sDNA screening cascade at a regional healthcare system in South Carolina, tracking outcomes at three sequential steps: test adherence, test positivity, and follow-up colonoscopy. Overall adherence was 67.9%, test positivity was 17.8% among adherent tests, and follow-up colonoscopy was completed by 69.9% of eligible positive patients within 1 year. Multivariable analyses identified insurance type, household income, patient outreach approach, prior test return history, and comorbidity as independent predictors of adherence, while income and race/ethnicity were independent predictors of time to follow-up colonoscopy. Together, these findings provide real-world insight into attrition across the CRC screening continuum and identify potentially modifiable patient- and system-level factors associated with screening completion.

The adherence rate of 67.9% is broadly consistent with estimates from prior real-world analyses of mt-sDNA adherence, which have reported return rates generally in the range of ~70%.^21–23^ These findings are also generally consistent with prior analyses conducted in other healthcare systems, including Beth Israel Lahey Health, Tufts Medical Center, and Hartford HealthCare, despite differences in patient demographics, payer mix, and healthcare delivery settings.^21–23^ The steep initial rise in the cumulative adherence curve, with more than 56% of eligible tests returned within the first 30 days, suggests that early patient response is the dominant pattern among adherent individuals.

Insurance type was the strongest demographic predictor of adherence in multivariable analysis. Medicaid-insured patients had substantially lower adherence than those with commercial or Medicare-type coverage (49.9% vs 68.7%-74.1%), and this disparity persisted after adjustment for income, age, and other covariates. These findings are consistent with a body of evidence demonstrating that Medicaid-insured and low-income populations face greater structural and logistical barriers to preventive care uptake.^24,25^ Notably, however, Medicaid patients are more likely to adhere to mt-sDNA testing than to FIT.^17^ The independent income gradient observed in this study, with odds of adherence approximately 35% higher in the highest income group than the lowest, further underscores the socioeconomic patterning of screening completion at the health system level. Targeted patient navigation, reminder programs, and barrier-reduction interventions for lower-income and Medicaid-insured patients may represent priority strategies for improving overall adherence.

The association between digital outreach and adherence was among the strongest findings in the logistic regression model. Full digital outreach was associated with more than twice the odds of adherence compared with no digital outreach (aOR, 2.74), and SMS-only outreach also significantly increased the odds of adherence (aOR, 1.53). These findings align with a growing evidence base suggesting potential benefit from technology-mediated patient engagement in CRC screening programs.^26,27^ Patient navigation systems (including Spanish language navigation, which improves adherence in Spanish-speaking populations) are built into mt-sDNA testing to encourage screening adherence.^28^ Prior test return history was an equally strong predictor of adherence (aOR, 2.01), suggesting that patients who have previously engaged with mt-sDNA screening are significantly more likely to repeat the behavior. This finding suggests that prior screening engagement may be an important marker of future screening participation. The large “unknown” outreach category (28.8% of tests), which showed unexpectedly high adherence and positivity rates in unadjusted analyses, limits interpretation of outreach-related findings and warrants further investigation; its interpretation is limited until the nature of the missing outreach data is clarified, and this represents a notable data quality consideration for the health system.

The test positivity rate of 17.8% is broadly consistent with the 15% positivity rate reported in a prior real-world mt-sDNA analysis.^29^ The higher rate observed here likely reflects the age and comorbidity profile of this cohort, which is dominated by Medicare Advantage patients (57.7%) and skewed toward older age groups. Positivity increased markedly with age in this study, from 4.8% at 45-49 years to 21.5% at 75-85 years, consistent with the known age-related increase in prevalence of colorectal neoplasia.^30^

The follow-up colonoscopy rate of 69.9% within 365 days of a positive result is within the range reported in other real-world analyses of mt-sDNA screening completion, which have found rates ranging from ~60% to ~75% depending on population and coverage type.^16,17^ Most demographic and clinical characteristics were not independently associated with time to follow-up colonoscopy after multivariable adjustment. The income gradient, however, was a notable exception; patients in the lowest income group had a follow-up colonoscopy rate approximately 12 percentage points below those in the middle-income group (61.8% vs 73.5%), and completed follow-up at a significantly slower rate after multivariable adjustment. Financial barriers historically associated with diagnostic colonoscopy, including cost-sharing requirements that apply when a colonoscopy follows a positive screening test, may contribute to this disparity.^31^ Currently, the Affordable Care Act prohibits payers from implementing cost-sharing for follow-up colonoscopies, which may lessen the financial barriers to CRC screening completion.^31^

To evaluate the potential influence of restricting the primary analysis to patients with continuous post-result insurance enrollment, we performed a sensitivity analysis including all positive mt-sDNA tests regardless of enrollment continuity. The observed follow-up colonoscopy completion rate remained similar (68.2% vs 69.8% in the primary analysis), suggesting that the continuous enrollment requirement had little impact on the overall estimate and supporting the robustness of the primary findings.

This study has several limitations. The single-health-system design at a regional health system in South Carolina may limit generalizability to health systems with different patient demographics, insurance distributions, or outreach program structures. Spartanburg Regional Healthcare System serves a population characterized by substantial Medicare Advantage enrollment and a predominantly low-to-middle-income patient population, and findings may not fully reflect screening patterns in other healthcare settings. As an integrated regional delivery network, Spartanburg may differ from national reference populations, from academic medical centers with more specialized referral patterns and research infrastructure, and from smaller community practices with different payer mixes and outreach capabilities; these structural differences should be considered when extrapolating these findings to other settings.

Treating each test kit as an independent observation means that patients contributing multiple tests may exert disproportionate influence on certain estimates. Because prior return history was associated with higher adherence, inclusion of repeat testers may have modestly increased overall adherence estimates compared with a strict patient-level analysis. Residual confounding from unmeasured socioeconomic, behavioral, and healthcare access factors may also have influenced screening participation and follow-up completion. The large “Unknown” outreach category represents a potential source of misclassification, and its unexpectedly high adherence association limits interpretation of outreach-related findings. Sparse cell sizes for several subgroups, including obstetrician/gynecologist providers (n = 94), partial digital outreach – email-only group (n = 31), and Traditional Medicare patients (n = 116), precluded their inclusion in regression models or resulted in wide CIs; findings in these groups should be interpreted accordingly. The primary follow-up colonoscopy analysis was restricted to patients with ≥365 days of post-result insurance coverage to ensure complete ascertainment of claims. However, a sensitivity analysis including all positive mt-sDNA tests regardless of enrollment continuity yielded a similar follow-up colonoscopy completion rate, suggesting that this restriction had minimal impact on the overall findings. Nevertheless, incomplete claims capture among patients who lost insurance coverage remains a potential source of bias. This study does not capture downstream outcomes beyond follow-up colonoscopy, including histopathological findings, CRC diagnosis, or mortality. The requirement for continuous insurance enrollment excluded a substantial proportion of initially identified tests and may limit generalizability to populations with less stable insurance coverage.

Strengths of the study include the characterization of all three cascade steps within a single real-world cohort, enabling a longitudinal view of attrition across the screening pathway. The availability of outreach type and prior return history data, which are not routinely available in large national datasets, provides novel insights into potentially modifiable system-level predictors of adherence. The extended study period (January 2016–June 2023) provides an adequate sample size to support multivariable analyses and captures real-world practice patterns over multiple years of program operation. These findings may help inform targeted quality improvement initiatives aimed at improving completion of stool-based CRC screening pathways in community healthcare settings.

## CONCLUSIONS

In this real-world study of mt-sDNA screening within a regional healthcare system, approximately two-thirds of eligible test kits were returned and nearly 70% of positive tests eligible for follow-up assessment completed follow-up colonoscopy within 1 year. Adherence and follow-up completion were associated with several patient- and system-level factors, including insurance type, household income, digital outreach, and prior screening participation. These findings highlight opportunities for targeted interventions and quality improvement efforts aimed at improving completion of stool-based CRC screening pathways, particularly among lower-income and Medicaid-insured populations.

### Disclosures

S.G., M.O., A.K., J.K., and M.G. are employees of Abbott and own stock or stock options in Abbott. A.H. and A.A. report no conflicts of interest.

### Ethical Approval

This secondary analysis of de-identified data was exempt from institutional review board oversight under 45 CFR §46.104(d)(4), consistent with HIPAA §164.514, and was conducted in accordance with the Declaration of Helsinki. Accordingly, the requirement for informed consent was waived. The analytic dataset provided to investigators contained only de-identified records.

## Supporting information

Online Supplementary Material

## Data Availability

The data underlying this study are not publicly available because of licensing and privacy restrictions but may be made available from the authors upon reasonable request and with appropriate permissions.
