# Supplementary material for: Real-World Adherence and Follow-Up Colonoscopy After Multitarget Stool DNA Screening in a Regional Healthcare System
Source: J Health Econ Outcomes Res. 2026 Sep 9;13(2):94–103. doi: 10.36469/001c.168717 (PMC13602028; doi:10.36469/001c.168717)
Supplement: Supplementary file 1 [file jheor_2026_13_2_168717_364796.pdf]

Journal of Health Economics and Outcomes Research

Online Supplementary Material

Real-World Adherence and Follow-up Colonoscopy After Multitarget Stool DNA Screening in a Regional Healthcare System. *JHEOR*. 2026;13(2):94-103. doi:10.36469/jheor.2026.168717

Table S1: Sensitivity Analysis of Follow-up Colonoscopy Completion

This supplementary material has been provided by the authors to give readers additional information about their work.

This is an open-access article distributed under the terms of the Creative Commons Attribution 4.0 International License (CCBY-4.0). View this license's legal deed at http://creativecommons.org/licenses/by/4.0 and legal code at http://creativecommons.org/licenses/by/4.0/legalcode for more information.

Table S1. Sensitivity Analysis of Follow-up Colonoscopy Completion[a]

**Analysis**	**Denominator**	**Completed Follow-up Colonoscopy, n (%)**
Primary analysis (≥365 days continuous post-result insurance enrollment)	853	596 (69.9)
Sensitivity analysis (all positive mt-sDNA tests regardless of post-result continuous enrollment)	1018	694 (68.2)

Abbreviation: mt-sDNA, multitarget stool DNA.

[a]The primary analysis required continuous health insurance enrollment for 365 days following a positive mt-sDNA result to ensure complete ascertainment of follow-up colonoscopy claims. The sensitivity analysis included all positive mt-sDNA tests regardless of post-result enrollment status and evaluated observed follow-up colonoscopy claims within 365 days after the positive test result.
